# Objective-Free Ultrasensitive Biosensing on Large-Area Metamaterial Surfaces in the Near-IR

**DOI:** 10.1021/acsami.4c04777

**Published:** 2024-06-13

**Authors:** Nurten Koc, Ali Belarouci, Evren Oktem, Serap Aksu

**Affiliations:** † Materials Science and Engineering, Koc University, Istanbul 34450, Turkey; ‡ Univ Lyon, ECL, INSA Lyon, CNRS, UCBL, CPE Lyon, INL, UMR5270, Ecully 69130, France; § Biomedical Science and Engineering, Koc University, Istanbul 34450, Turkey; ∥ Department of Physics, Koc University, Istanbul 34450, Turkey

**Keywords:** plasmonic metamaterial perfect absorbers, laser interference lithography, refractive index-based biosensing, real-time protein binding, lens-free spectroscopy

## Abstract

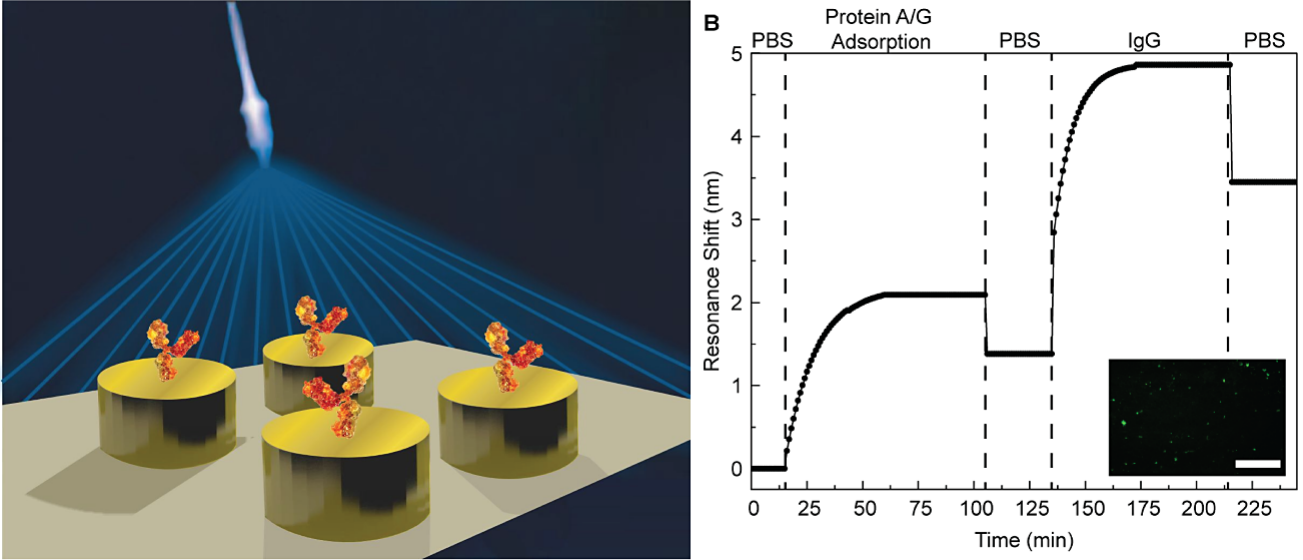

Plasmonic metamaterials have opened new avenues in medical diagnostics. However, the transfer of the technology to the markets has been delayed due to multiple challenges. The need of bulky optics for signal reading from nanostructures patterned on submillimeter area limits the miniaturization of the devices. The use of objective-free optics can solve this problem, which necessitates large area patterning of the nanostructures. In this work, we utilize laser interference lithography (LIL) to pattern nanodisc-shaped metamaterial absorber nanoantennas over a large area (4 cm^2^) within minutes. The introduction of a sacrificial layer during the fabrication process enables an inverted hole profile and a well-controlled liftoff, which ensures perfectly defined uniform nanopatterning almost with no defects. Furthermore, we use a macroscopic reflection probe for optical characterization in the near-IR, including the detection of the binding kinematics of immunologically relevant proteins. We show that the photonic quality of the plasmonic nanoantennas commensurates with electron-beam-lithography-fabricated ones over the whole area. The refractive index sensitivity of the LIL-fabricated metasurface is determined as 685 nm per refractive index unit, which demonstrates ultrasensitive detection. Moreover, the fabricated surfaces can be used multiple times for biosensing without losing their optical quality. The combination of rapid and large area nanofabrication with a simple optical reading not only simplifies the detection process but also makes the biosensors more environmentally friendly and cost-effective. Therefore, the improvements provided in this work will empower researchers and industries for accurate and real-time analysis of biological systems.

## Introduction

The innovative plasmonic metallic nanoantennas have emerged as a cutting-edge technology at the forefront of modern photonic applications in the near-IR frequencies.^1−3^ The 3D engineering of metallic nanostructures enables metamaterial surface (MS) that fully absorbs the incident light and open new avenues for medical diagnostics,^4−6^ by providing real-time, label-free detection, and quantification of various analytes with exceptional sensitivity,^7,8^ specificity,^9^ and rapid response times.^10^ Label-free detection capability of these nanostructures provides significant advantages in the detection process. However, the cost of nanofabrication, even for sub-mm^2^ areas, and the need for microscope-based bulky optical signal readers from such small areas limit their adaption in clinics or resource-limited settings. Hence, the large area nanopatterning of metallic nanostructures is a major step to eliminate the need for complicated optics for widespread adoption of label-free optical sensing in limited infrastructure settings.

One other challenge to adapt the large area MS in the diagnostics market is the need for a scalable and repeatable nanopatterning method, as the same sensor is to be manufactured many times. The electron beam lithography (EBL) provides remarkable resolution for nanopatterning and has been the gold-standard method for metallic nanostructure manufacturing.^11^ However, it is limited by scalability and strives to cover several square centimeters in a cost-effective way. Various smart approaches have been suggested to eliminate those limits.^12−14^ Each of them offers a distinctive strength and can enable complex-shaped nanostructures either on planar or on flexible surfaces.^15^ The ability to fabricate complex structures is crucial for photonics advancements. On the other hand, antennas with simple geometry (circular, triangular, or square) that are periodically placed on conventional substrates have been reported to provide ultrasensitive detection of medically relevant biomolecules.^1,6,16^ Laser interference lithography (LIL) that has long been used for fabrication of 1D optical gratings also represents an alternative approach that is suitable for the patterning of periodic 3D submicron simple geometry structures with controlled size and shape.^17,18^ LIL exploits the recording of an interference field pattern into a photoresist and, in combination with liftoff or physical etching steps, can be used for the fabrication of arrays of defect-free metallic nanostructures with fine-tuned optical response. Even though LIL is limited for shape flexibility and fine-tuning of the size, once the interference set up is tuned, LIL offers the advantage of mass production and provides wafer-scale manufacturing scheme.^19,23^

In this work, we manufacture a light-absorbing metamaterial surface (MS) over a large area (4 cm^2^) that operates in near-IR frequencies by using laser interference lithography. The optical characterization of the fabricated MS and the sensing protocols are performed without a microscope, using an objective-free reflection probe, that can promote the use of the MS in resource-limited settings. The MS is composed of a multilayer metal–insulator–metal configuration, where a thin (30 nm) SiO_2_ spacer is sandwiched between an optically thick (150 nm) gold bottom layer and periodically patterned circular nanodisc (radius: 160 nm)-shaped gold antenna on the top layer. The simulation results reveal that the designated metamaterial surface demonstrates perfect absorption (reflection ∼2%) with a narrow bandwidth (∼30 nm) and a high-quality factor (*Q* = λ/bandwidth, ∼ 25) resonance. The fabricated nanodisc antennas on the top are well-defined and have vertical side profiles. They are uniformly patterned with almost no defects over the whole sensor area. The radius size distribution is very uniform with ±6 nm deviation on ∼160 nm nanodisc radius. The optical measurements obtained from different points on the same sensor chip give nearly identical optical responses with <1% resonance wavelength deviations, which proves the homogeneity of the nanofabrication over large area. The sensing capacity of the MS is characterized using immunologically relevant proteins of recombinant protein A/G and immunoglobin G,^20^ where their binding kinetics are displayed in real time. The alteration in the sensor microenvironment leads up to a 685 nm resonance wavelength shift per refractive index unit change, which is on par with the EBL-fabricated surfaces.^16^ Hence, utilizing LIL for the large area nanopatterning of MS can pave the way from laboratory-based research to the portable point-of-care device market.

**Figure 1:**
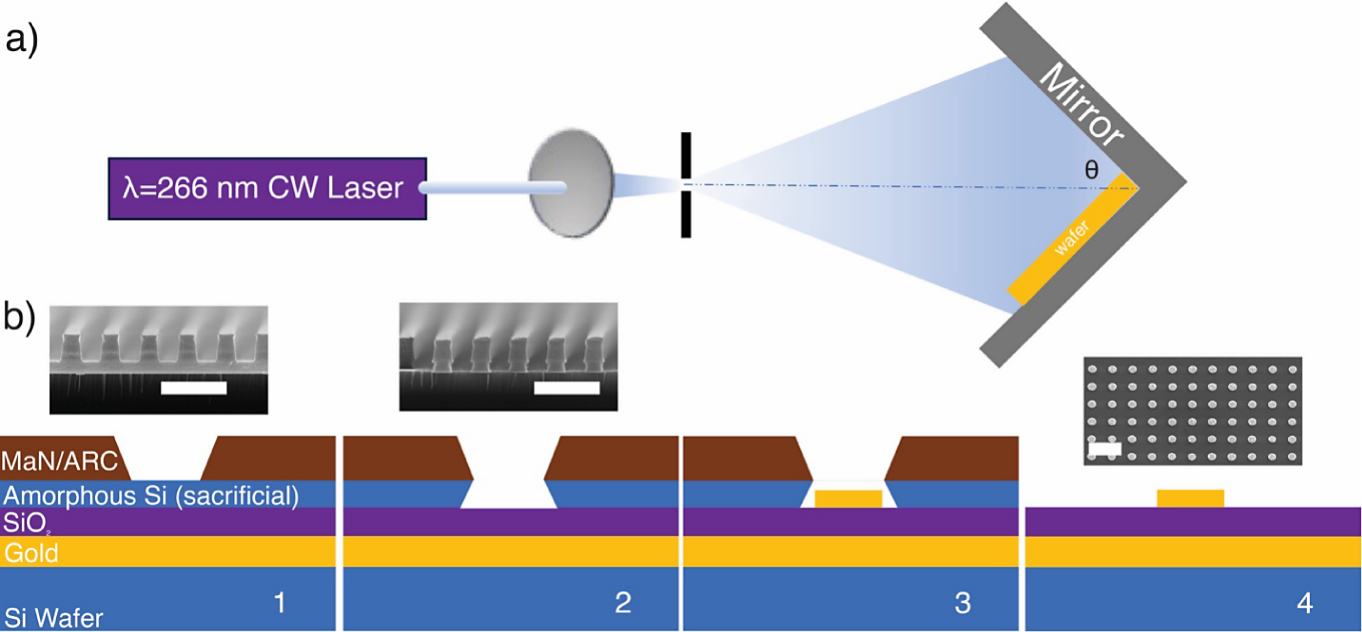
Illustrations of the laser interference set up and the fabrication steps. (a) The distance between the continuous wave laser and the mirror is 2 m. Periodicity of the interference can be changed by tuning the angle between the incident light and the mirror (θ). (b) The cross-section illustration of the fabrication steps, with corresponding SEM images on top. 1: the application of the laser interference and development of the resist; 2: etching of the sacrificial layer to obtain an inverted wall profile for a controllable lift off; 3: deposition of gold with a Ti sticking layer on silica; 4: KOH etching of the sacrificial layer enables smooth liftoff. The scale bar on all SEM images is 1 μm.

There are two principal configurations for LIL: the Lloyd’s mirror interferometer used for high resolution and the dual beam interferometer, used for large areas. In our experiment, the exposure is carried out using Llyod’s mirror setup,^21^ which is less sensitive to fluctuations and easier to align than the dual beam interferometer (Figure 1a). Typically, multiexposures are utilized to enable multidimensional periodic gratings. Recent efforts have been mostly focused on generating 2D gratings with a single exposure to solve groove depth difference problems inherent in the double exposure techniques.^22^ Similarly, fine-tuning the exposure dose profiles combined with grayscale patterned secondary exposure can enable perfectly spatially controlled groove line widths at wafer scale.^23^ Some other approaches use a plasmonic mask to transfer the patterns^24^ or use a spatial light modulator involved pulser laser to directly transfer the pattern with ablation on the substrate.^25^ Here, we keep the laser interference set up as simple as possible using only a Lloyd mirror. Instead, we introduce a sacrificial layer (amorphous Si) to minimize the effect of the groove on the resulting nanostructure quality by eliminating the photoresist lift off process. For LIL setup, a 10 mW, 266 nm UV laser is used as a light source. A spatial filter with an UV objective lens and a 10 μm diameter pinhole allows the high-frequency noise to be removed from the beam to provide a clean Gaussian profile. The diverging beam travels 2 m over an optical table to a Lloyd’s mirror interferometer mounted on a rotation stage. The portion of the beam that is reflected off the mirror surface interferes with the part of the beam that is directly incident on the sample. The interference leads to a standing wave intensity distribution in the photoresist with a period *P* = λ/(2sin θ), where λ is the wavelength of the laser beams and θ the half angle at which two beams intersect. The period can therefore be adjusted by changing the θ angle of the rotation stage. After the resist is developed, parallel stripes on the sample surface are present. Square array-like patterns can be obtained using a double-exposure technique, where after the first exposure, the substrate is rotated by 90° and is exposed again. The 2 m distance between the mirror and laser can pattern an area of 5 × 5 cm in our setup. Given that the coherence length of the laser used is above 20 m, patterning a 4 in. wafer scale nanofabrication is also possible by simply enlarging the distance between the laser and the Lloyd mirror.

## Experimental Section

**Figure 2:**
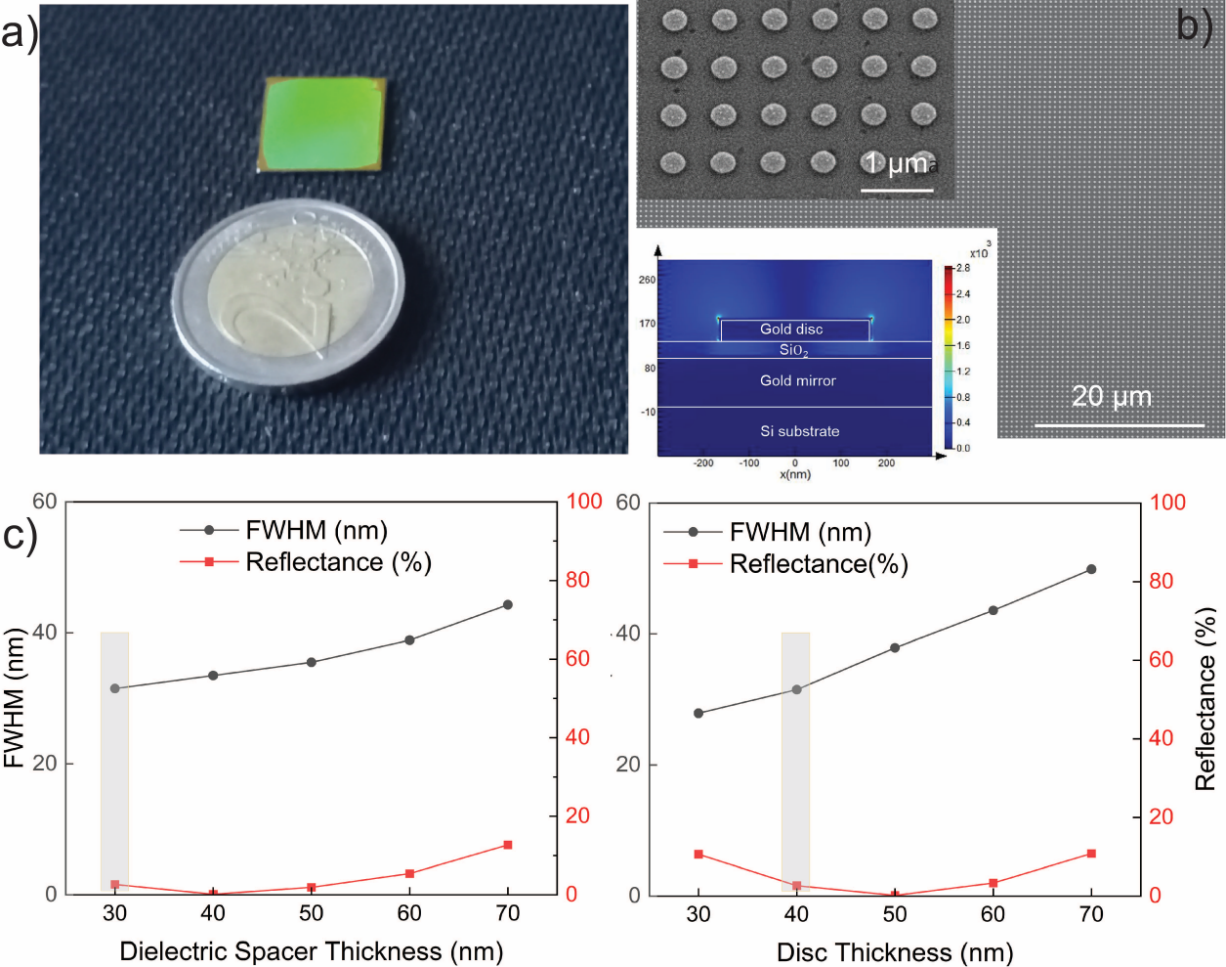
Large area 3D metamaterial perfect absorber surface is fabricated by using laser interference lithography. (a) The photograph of a typical sensor shows the uniformity over the whole sensor area. (b) SEM images also confirm the homogeneity of the nanodisc sizes over all area. Inset shows the simulated near field electric field enhancement on a nanodisc. The enhanced field intensity between nanodisc and air is on the order of 10^3^. (c) On the left, the simulation results show the effect of the SiO_2_ spacer thickness on the reflectance and bandwidth. Similarly, on the right, the effect of the gold nanodisc thickness on the reflectance and bandwidth is shown. The optimum thicknesses are decided in the shaded region by considering the minimum reflection and fwhm.

The preparation of the 3D metamaterial surface starts by cutting a Si (100) 4 in. wafer into 2 cm × 2 cm chips and cleaning them in acetone and isopropanol for 5 min in an ultrasonic bath, followed by blow drying with nitrogen. A 5 nm-thick titanium layer (Ti, adhesion layer), a 150 nm-thick gold layer, and a 2.5 nm-thick titanium layer are deposited by e-beam evaporation followed by a 30 nm-thick silica spacer layer and a 160 nm-thick amorphous silicon sacrificial layer deposited by PECVD (Figure 1b). The reasoning behind the deposition of the sacrificial layer is to facilitate the liftoff process to enable smooth, well-defined wall profiles for the nanodiscs over the whole area in a more controllable way. The 2.5 nm titanium layer is used as an adhesion layer for the silica spacer to stick to the bottom gold mirror. This sticking layer is particularly important for the real-time sensing measurements, as without a sticking layer, the dielectric layer comes off from the substrate in water. Afterward, a 300 nm-thick MaN-2403 (Micro resist technology GmbH) negative resist is spin-coated and baked at 90 °C for 1 min. To prevent back reflection at the sample-resist interface, a 200 nm-thick antireflection coating layer (ARC: AZ-BARLi-II 90, MicroChemicals) is coated underneath the photoresist layer. The sample is then exposed in the Lloyd’s mirror type LIL setup with a double exposure by the 90° sample rotation after the first exposure. The exposure times are on the order of minutes, depending on the designated hole size, providing fast and high throughput nanofabrication. Increasing the laser power can further minimize the exposure time.^19^ The unexposed photoresist is further removed by proper development, leaving behind a homogeneous mask with arrays of cylindrical holes. The pattern is subsequently transferred to the ARC layer by ICP-RIE in O_2_/Ar. Isotropic etching of amorphous silicon is then performed under SF_6_/O_2_ gas (SF6:100 sccm, O_2_: 20 sccm, power = 20 W) to provide an inverted profile. A 40 nm-thick gold layer with a 5 nm-thick titanium adhesion layer is then deposited by e-beam evaporation. Finally, the liftoff process is implemented in a 34% potassium hydroxide solution at 70 °C. Figure 1b displays all the mentioned procedures from steps 1 to 4, with the SEM images taken from cross-section. The SEM images confirm the success of using the sacrificial layer that provides an inverted hole profile, leading to a more controllable liftoff process, which enables a very uniform, almost defect-free metamaterial surface over 4 cm^2^ area at the end (Figure 2a,b).

## Results and Discussion

A variety of simulations are performed to optimize the geometrical dimensions of the 3D metamaterial surface (MS) to deliver the maximum absorption. To calculate the near- and far-field optical properties, the proposed MS unit cell is simulated by using commercially available software (Lumerical Inc.). The finite-difference time-domain (FDTD) method is employed for calculations. As the MS is composed of periodic nanodiscs, only a single unit cell is simulated by applying proper boundary conditions in all directions. Unpolarized plane waves are incident normally from the top along the *z*-axis, mimicking the experimental setup. Periodic boundary conditions are imposed along both the *x* and *y* directions, while perfectly matched layers are used along the propagation direction (*z*-axis). The absorption of the system is calculated using the relation absorption = 1 – reflection, as the transmission is zero due to optically thick gold film on the bottom. As discussed in our previous report,^6^ the resonance wavelength strongly depends on the periodicity and not significantly affected by the diameter of the nanodisc (Figure S1a). This indicates that the measured and simulated resonances occur due to the excitation of the propagating surface plasmons.^26^ The electric field intensity is predominantly confined around the top edges of the nanodisc, as shown in Figure 2b, specifically on the disc–air interface, rather than being concentrated on the disc-dielectric interface. As a result, the resonance is strongly associated with periodicity (Figure S1a). However, it is important to note that the radius plays a significant role in a different resonance mode at larger wavelengths (Figure S1c), which possesses localized surface plasmon (LSP) characteristics. We have previously showed that to minimize the reflection, it is crucial to study the scattering properties of the individual nanodiscs, which are operating as nanoantennas.^6^ The precise selection of the SiO_2_ spacer thickness plays an important role to achieve the so-called critical coupling condition, where the radiative and intrinsic (nonradiative) damping rates become identical.^27^Figure 2c shows the effect of SiO_2_ thickness and the nanodisc thickness on perfect absorption. To choose the optimum thicknesses, we consider that the resonance must show minimum reflection to ensure the perfect absorption. At the same time, resonance must have a minimum full width at half-maximum (FWHM). Figure 2c shows that when the SiO_2_ thickness is 40 nm, we get the minimum reflection at the expense of a larger FWHM. Thus, we choose to use a 30 nm SiO_2_ thickness that still gives <2% reflection together with a high-quality resonance with minimum FWHM. A similar discussion also goes for the nanodisc thickness. In addition, keeping the spacer thickness at 30 nm, we choose the disc thickness to be 40 nm, which still gives the perfect absorption at minimum FWHM. The optimum thicknesses are depicted with a shaded region in Figure 2c. The results indicate that there is a critical SiO_2_ thickness (30 nm) and gold nanodisc thickness (40 nm) that yields the maximum absorption with the smallest full width at half maxima line width. The electric field concentrated on the antenna surface at the resonance wavelength indicates a thousand-fold enhancement of the incident light intensity, which is consistent with our prior reports^6^ (Figure 2b). Furthermore, our simulations indicate that the disc size is not only instrumental for fine-tuning the resonance wavelength but also an important parameter to generate the perfect absorption. Previously, we showed that at the critical coupling regime that leads to the perfect absorption, absorption and scattering cross sections can be much greater than the geometrical cross section, and the absorption cross section is almost identical to the scattering cross section.^6^ Enlarging the nanodisc also enlarges the scattering cross section and thus must affect the perfect absorption. Our findings reveal that tuning the nanodisc radius from 100 nm to 160 affect the perfect absorption by minimizing the reflection (Figure S1b). Thus, we choose to work with nanodiscs of 160 nm radius and 700 nm period that give resonances at ∼750 nm wavelength with minimum reflection. This configuration is also suitable for sensing experiments in water, where the resonance shifts to ∼950 nm wavelength, which is still measurable using a standard Si based spectrometer.

Once the 3D MS with the optimum optical property is designated, the MS is fabricated using laser interference lithography. LIL is a very reliable and repeatable method; however, it can be performed for a certain periodicity and size range depending on the laser wavelength used. The periodicity of the resulting pattern is determined by the wavelength of the laser light and the angle at which the beams intersect (*P* = λ/(2sin θ)). The achievable periodicity is typically no smaller than half of the wavelength of the light used. With our setup, the periodicity *P* can be easily adjusted from almost 1 μm down to 150 nm, by changing the angle θ of the rotation stage. Besides, the dimension of the nanostructures can be controlled by using different exposure doses (i.e., time of exposure) of the photoresist within a range of sizes between 30% (20 min exposure) and 70% (2 min exposure) of the periodicity. The fabricated structure size is therefore limited by the achievable period.

**Figure 3:**
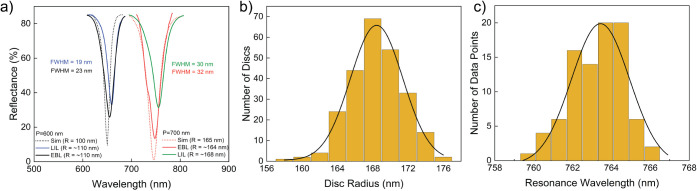
Optic and metric characterization of high quality LIL-fabricated nanodisc antennas. (a) The reflectance comparison of nanodics fabricated by LIL vs EBL. The red-dashed line shows the simulation results for nanodiscs with a period of 700 nm, radius of 160 nm, and the blue-dashed line shows simulation results for nanodiscs with a period of 600 nm, radius of ∼100 nm. Red-dashed curve shows a near unity absorption. EBL- and LIL-fabricated structures show similar FWHM, resonance frequency, and intensity. (b) The radius distribution of the LIL-fabricated nanodiscs is shown. The size deviations are ∼ ± 6 nm, presenting the homogeneity over a large area. (c) Hundreds of reflection measurements are performed covering the whole area on a large area metamaterial surface. The optical deviation is less than 1%.

We first test the optical quality of the LIL-fabricated nanodisc array by comparing it against an electron beam-fabricated one (Figure 3a). The EBL-fabricated nanostructures are written on a poly methyl methacrylate (PMMA) resist bilayer over a 5 × 5 mm area using a Raith EBPG 5000 system at 100 keV. The writing took 6 h 30 min with EBL, where it is a matter of minutes with LIL for a 2 × 2 cm area. The reflection measurements of EBL- and LIL-fabricated structures are given in Figure 3a accompanied by the simulation results (dashed lines). The reflection measurements are performed using a reflection probe (Filmetrics F20, KLA) that sends the incident light and collects the reflected light from the sensor surface with a single optical fiber. The reflection probe is connected to a spectrometer and light source. A gold mirror is used to obtain the reflection background. The reflection probe illuminates the surface vertically and collects the reflected spectrum. The spot size of the incident light is 1.5 mm. All spectra are baseline corrected, and the raw data is available in Figure S2. Simulations show the perfect absorption on the MS (reflection <2%) at certain wavelengths. The LIL-fabricated MSs show similar resonance profile and similar FWHM with the simulations; however, unlike the simulation results, LIL- and EBL-fabricated MSs do not show close to zero reflection. This discrepancy is due to the need of a sticking layer (2.5 nm Ti). The measurements in water flow necessitate the application of a sticking layer as the SiO_2_ spacer lifts off from the bottom gold mirror in water flow (Figure S3). However, the use of a sticking layer below SiO_2_ comes with a trade-off: damping the absorption and increasing the reflection^28,29^ and causes a deviation from the simulation results (Figure 3a). Such a sticking layer problem is also evident in perfect absorbers operating in the mid-IR,^4,5^ but the effect on the reflection intensity is more notable in the near-IR. The full width at half-maximum for EBL-fabricated structures is measured as 23 nm, where the LIL-fabricated ones have FWHM of 19 nm for nanodisc with *P* = 600 nm and *R* = 110 nm. Figure 3a shows that the resonance quality of LIL-fabricated nanodiscs is similar to that of EBL-fabricated ones in terms of line width and resonance frequency for different periods and disc radius. Small deviations could be attributed to the minimal nonuniformity of the nanodiscs and the small variations in the sticking layer thickness. Achieving uniformity and maintaining precise control of the interference pattern over a larger area is a challenging task for LIL.^30 −33^ As mentioned before, our LIL setup is designed for wafer scale patterning. Highly homogeneous patterns can be produced over large areas, and the use of a relatively smaller area in this work ensures the homogeneity of the nanodiscs. To demonstrate the homogeneity, we present the radius size distribution of the LIL-fabricated ones for nanodisc with ∼160 nm radius. The distribution is calculated from multiple SEM images (e.g., Figure 2b inset) using ImageJ software. The maximum deviation from the average radius size on the sensor area is ±6 nm (∼4%) for LIL-fabricated discs (Figure 3b), where it is ±3 nm for EBL-fabricated ones (∼3%, Figure S4). This result proves the EBL-like homogeneity of the LIL-fabricated structures. Data showing the homogeneity of size distribution using a larger number of discs fabricated with LIL is available in Figure S5. Moreover, we perform reflection measurements from hundreds of different points on the same sensor surface. Figure 3c shows that the resonance wavelength distribution is again highly uniform over the whole sensor area, with less than 1% deviation. All the metric and optic characterizations show that the LIL-fabricated structures using a sacrificial layer enable high quality photonic structures as well as EBL writing but in low cost and large area manner.

**Figure 4:**
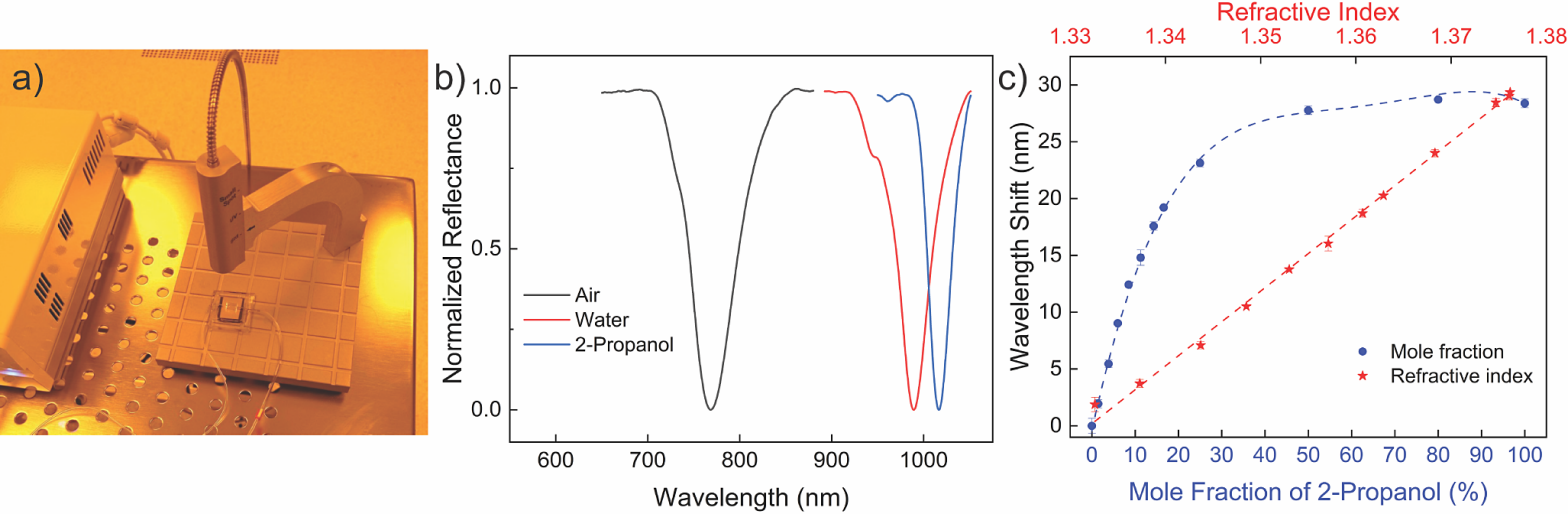
Sensitivity of the LIL-fabricated large area metamaterial surface is characterized in a microfluidic flow. (a) The photograph of the lens-free reflection probe and the flow conditions utilized for reflection measurements on sensor. The same set up is used for all optical characterization presented in this work. (b) The resonance shift from air to water and to 2-propanol. The resonance shift is >200 nm in water. (c) The sensitivity of the metamaterial surface against the refractive index change. The refractive index change is achieved by tuning the mole fraction of 2-propanol. A peak refractive index, thus the maximum resonance shift, is recorded for a mole fraction of 0.8.

The sensing capacity of the manufactured MS is first tested by changing the refractive index of the microenvironment around the sensor. The sensitivity of the MS sensor against the refractive index change is characterized with different solvents that have varying mole percentages in water. The solvents are flown on the sensor surface at a flow rate of 14 μL/min using a custom-made microfluidic cell, based on polydimethylsiloxane (PDMS), during the optical measurements (Figure 4a,b). We use water and 2-propanol (water *n* = 1.33, IPA = 1.37) with different mole fractions to tune the refractive index from 1 (air) to 1.37. The refractive index of the binary mixtures of alcohols and water is obtained from Belda and Herraez.^33^ We should note that the mole fraction does not linearly change with the refractive index. As shown in Figure 4c, a peak refractive index, thus the maximum resonance shift, is recorded for a mole fraction of *x* = 0.8 for 2-propanol, agreeing well with the literature.^33^ The sensitivity of the MS is correlated with the amount of resonance wavelength shift to the red as the refractive index of the solvent increases (*S* = Δλ/Δ*n*). The sensitivity of the manufactured MS is measured as 685 nm per one unit of refractive index change, which is calculated as *S* = 650 nm/RIU for EBL-fabricated plasmonic sensors for the same period and thus the same particle number (Figure S6). The competitive nature of the LIL-fabricated metasurface sensitivity is further compared against previously reported values in Table S1.

**Figure 5:**
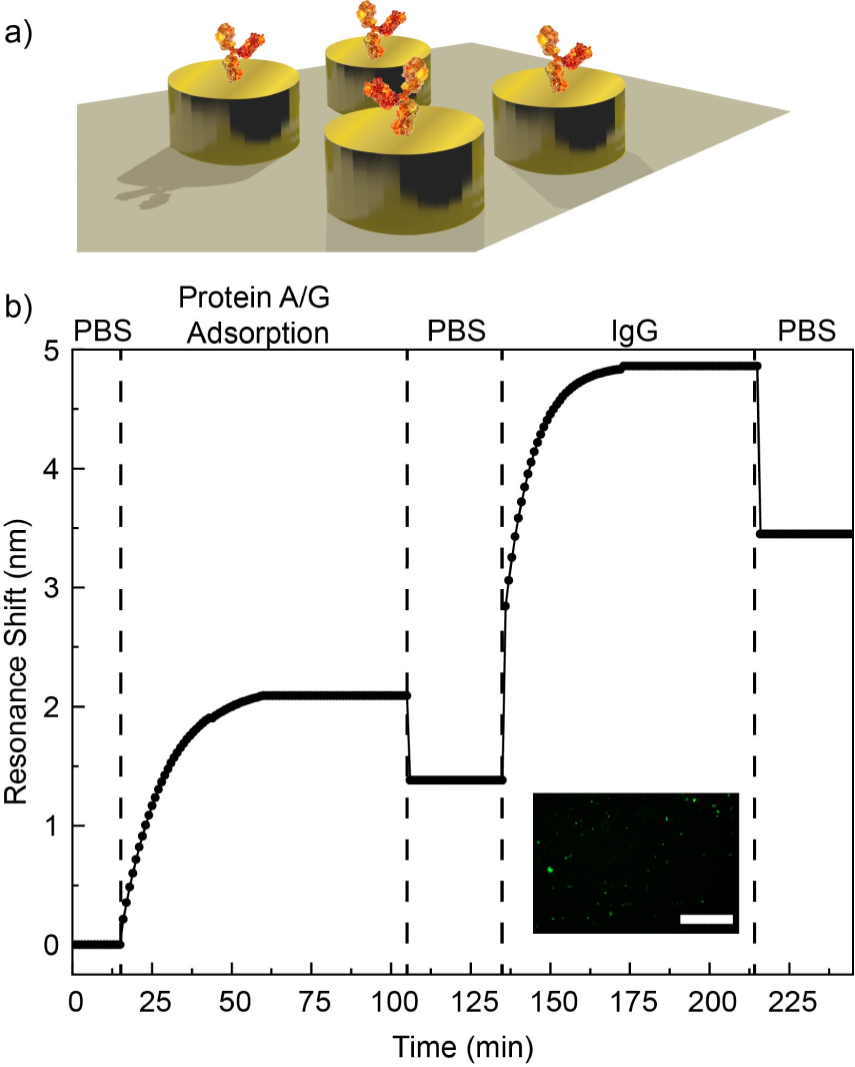
Time-dependent analysis of specific binding of IgG on protein A/G monolayers. (a) The physisorption of the protein A/G and IgG immobilization on gold nanodiscs. (b) The change on the resonance wavelength is recorded at 1 min intervals during protein A/G physisorption and IgG immobilization on it. The inset shows the fluorescence fingerprint of the FITC-labeled IgG on nanodisc, confirming the presence of the IgG on sensor. The scale bar displays 1 mm.

To demonstrate the real-time sensing capability of the LIL-fabricated low-cost metamaterial surface, we utilize recombinant protein A/G (Pierce) as a receptor for the specific binding of the immunoglobulin (IgG) (Figure 5a). The adsorption performance of the protein A/G and IgG on gold vs silica surface has been broadly investigated before.^20,34,35^ We used a custom-made microfluidic cell over the sensor to incubate the proteins. Between the measurements, the sensor surface is cleaned with phosphate buffered saline (PBS) solution. Before the tests, oxygen plasma is applied for a minute for cleaning and oxygenation of the surface, which helps with the physisorption of protein A/G on gold. First, PBS solution is applied on the sensor, and the reflection measurement is performed using the reflection probe (Figure 4a), without the need for a bulky benchtop microscope. After the surface was wetted with PBS, protein A/G with 500 μg/mL concentration in PBS is incubated on the sensor surface. The reflection measurements are taken at 1 min intervals. The resonance peak wavelength is acquired every minute, and the change on the wavelength is plotted as shown in Figure 5b. Even though protein A/G is a very small molecule with 50 kDa mass, we observe that a resonance shift in the first minute protein A/G is applied on the sensor. Figure 5b shows that after applying protein A/G for 45 min, the resonance shift reaches an equilibrium. This indicates that 45 min is the required time for protein A/G to be adsorbed fully on the sensor. Then the sensor surface is washed with PBS solution, where the shift drops as some of the protein A/G is gone from the vicinity of the nanodisc antennas. However, 1.5 nm resonance shift remains constant after 30 min of washing with PBS, which indicates that there is still protein A/G adsorbed on the gold surface. After washing, antimouse IgG (labeled with FITC) produced in goat (F5262, Sigma-Aldrich) with 50 μg/mL concentration is immobilized on the sensor surface, thanks to its affinity for protein A/G. Similar to the protein A/G, introducing IgG induces an immediate resonance shift, and the shift reaches a maximum plato after 45 min. Same washing conditions with PBS are again applied for 30 min after reaching the plato. The sensor surface is checked under a fluorescent microscope. The inset in Figure 5b shows fluorescence, which indicates the presence of IgG on the sensor area. We observe a 2 nm shift due to binding of the IgG on protein A/G. The shift with IgG is expected to be larger, as the biomass of the IgG is bigger compared to protein A/G. In Figure 5b, each black dot represents the average value of the resonance shift in consecutive 3 min, and the black line that connects the dots is an exponential fitting. The raw data showing the resonance wavelength shift versus time are available at Figure S7. Furthermore, after the protein binding events, the sensor can be cleaned by applying a piranha solution (H_2_SO_4_: H_2_O_2_, 3:1) for a minute. We observe a degradation of the optical quality of the sensor after three piranha cleanings (Figure S8). The overall results clearly show that the low-cost LIL-fabricated metamaterial surfaces are a prime candidate for time-dependent analysis of biomolecular bindings, or any refractive index change chemically happening on the surface.

## Conclusion

We demonstrate high-quality uniform nanopatterning of nanodisc antenna-based metamaterial surfaces using laser interference lithography. The introduction of a sacrificial layer provides excellent control over the sizes and enables controlled and well-defined nanostructures with 4% size deviation over all area, which is 3% for EBL-fabricated ones. This precise size control also brings a uniform optical quality over the whole area. The optical results show less than 1% resonance wavelength deviation, which proves that any point on the sensor has almost identical photonic properties. The optical characterizations are performed using a lens-free reflection probe, eliminating the need for bulky optics, which is the first necessary step toward miniaturization. The LIL-fabricated metamaterial surface experimentally shows a sensitivity of 685 nm/RIU, which is on par with EBL-fabricated ones and one of the highest reported. The large area sensor is used in the real-time sensing of protein A/G and IgG. A significant resonance shift is observed during the binding of low-mass receptor protein on sensors and the IgG monolayers on protein A/G, proving the quality of the sensor and the optics for real-time ultrasensitive measurements. Therefore, the use of the presented large area metamaterial sensors can pave the way for a truly hand-held ultrasensitive diagnostic device to be used at resource limited settings; and the modified LIL is a prime candidate for rapid and low-cost nanomanufacturing of such sensors.

## Supporting Information Available

Reflectance spectra of LIL-fabricated nanodiscs with different periods and varying radius; the effect of nanodisc radius on the resonance quality and experimental results, showing the effect of radius change on the second resonance mode (Figure S1); raw and baseline corrected spectra of LIL-fabricated nanodiscs (Figure S2); impact of sticking layer on the integrity of SiO_2_ thin film (Figure S3); SEM image and size distribution of EBL-fabricated nanodiscs (Figure S4); SEM images and size distribution of LIL-fabricated nanodiscs (Figure S5); reflectance spectra of EBL-fabricated nanodiscs with different periods in air, water, and 2-propanol (Figure S6); raw data acquired during the real-time sensing of protein A/G and IgG (Figure S7); effect of piranha cleaning on the metasurface, corresponding optical response and SEM images (Figure S8); and the sensitivity comparison of this work against previously reported values in literature (Table S1) (PDF)

## Abbreviations

MS: metamaterial surface
LIL: laser interference lithography
EBL: electron beam lithography
FWHM: full width at half-maximum
PDMS: polydimethylsiloxane
SEM: scanning electron microscope
PBS: phosphate buffered saline
UV: ultraviolet
IR: infrared
RIU: refractive index unit
PECVD: plasma enhanced chemical vapor deposition
