# Supplementary material for: Objective-Free Ultrasensitive Biosensing on Large-Area Metamaterial Surfaces in the Near-IR
Source: ACS Appl Mater Interfaces. 2024 Jun 13;16(25):32516–23. doi: 10.1021/acsami.4c04777 (PMC11212022; doi:10.1021/acsami.4c04777)
Supplement: Supplementary file 1 — am4c04777_si_001.pdf [file am4c04777_si_001.pdf]

Supporting Information

Objective free ultrasensitive biosensing on large-area metamaterial surfaces in the near-IR

*Nurten Koc[1], Ali Belarouci[2], Evren Oktem[3], Serap Aksu[1,3,4]**

[1]Materials Science and Engineering, Koc University, 34450 Istanbul, Türkiye.

[2]Univ Lyon, ECL, INSA Lyon, CNRS, UCBL, CPE Lyon, INL, UMR5270, Ecully 69130, France.

[3]Biomedical Science and Engineering, Koc University, 34450 Istanbul, Türkiye.

[4]Department of Physics, Koc University, 34450 Istanbul, Türkiye.

*Corresponding author: saksu@ku.edu.tr

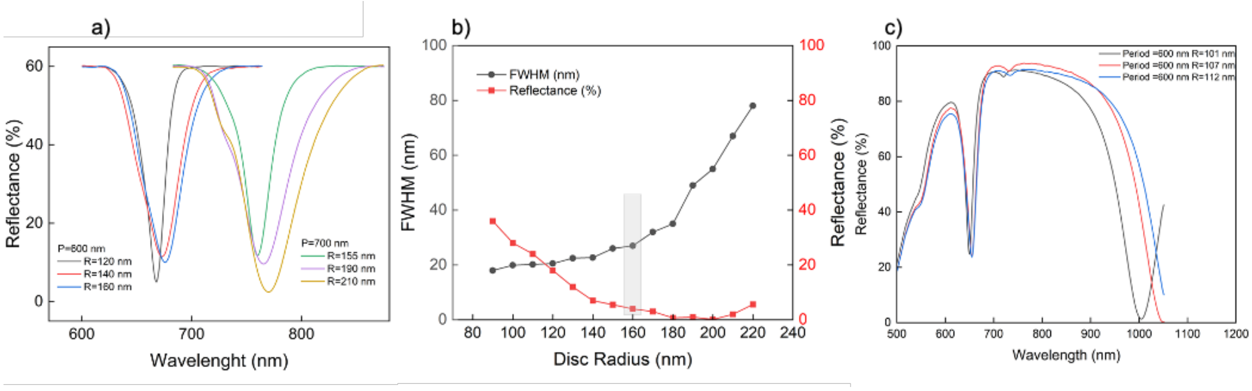

Figure S1. a) The reflectance of LIL fabricated nanodiscs with different period (P=600 and 700nm) and different radius R. The resonance wavelength is significantly relevant with the periodicity, confirming the propagating plasmon resonance. b) The simulation results reveal that tuning the nanodisc radius affects the perfect absorption by minimizing the reflection. When R~ 160nm, we get a high-quality resonance with optimum reflection and FWHM. c) The second resonance mode at far near-IR is significantly affected by radius change, confirming the localized surface plasmon resonance nature of the mode.

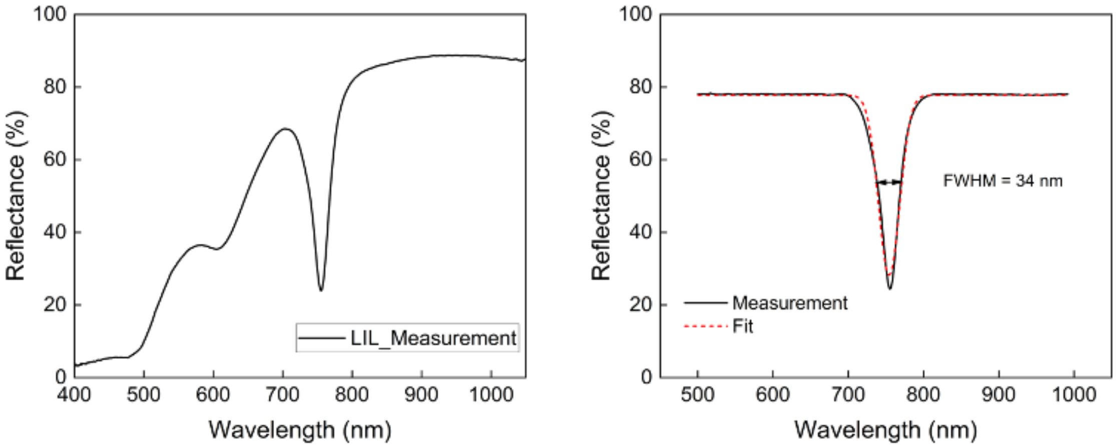

Figure S2. Raw data acquired from the spectrometer is shown on the left. The spectrum is baseline corrected on the right when needed.

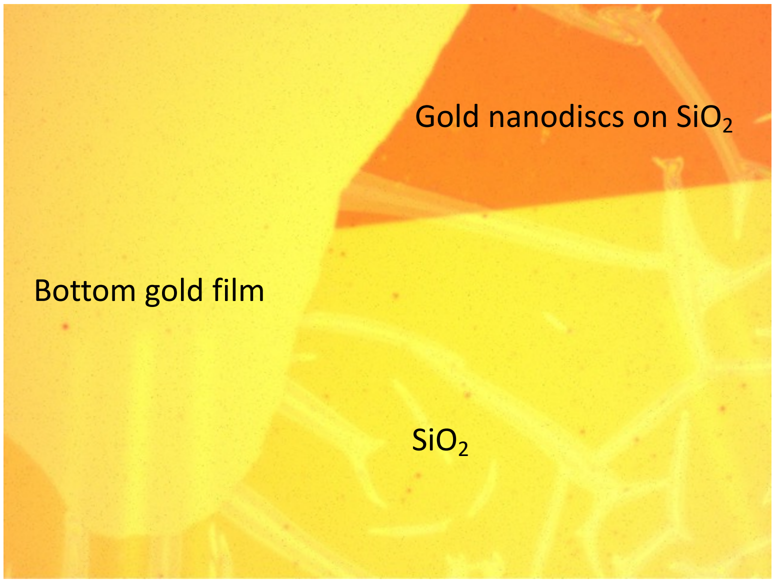

Figure S3. Forming a SiO_2 thin film on bottom gold film requires a sticking layer (2.5 nm Ti) in between. When that sticking layer is not applied, SiO_2 film cracks in water and comes off the surface with the nanodiscs on top. This sticking layer affects the reflection and dampens the perfect absorption.

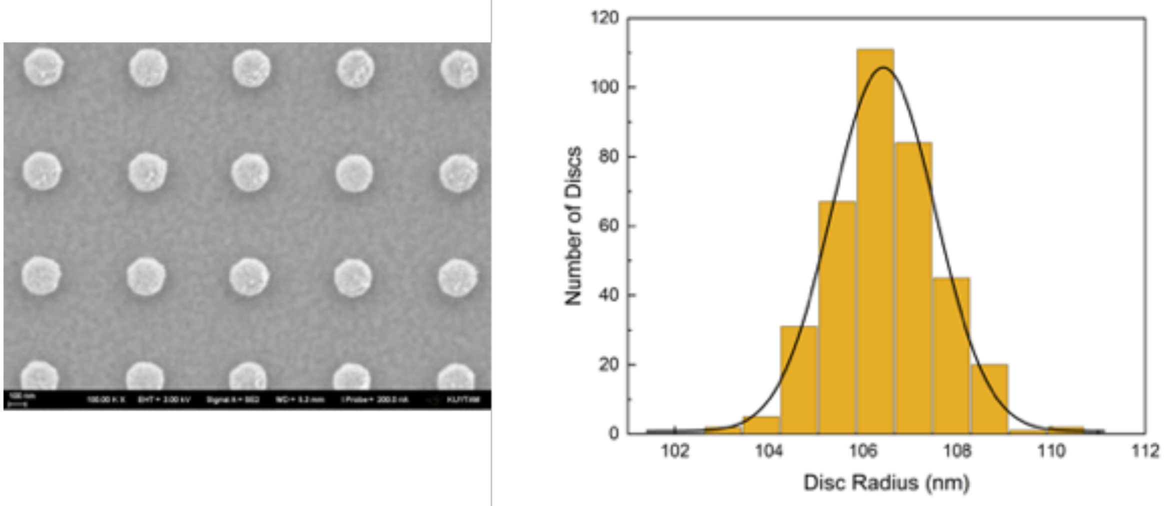

Figure S4. The SEM image on left shows the EBL fabricated metamaterial surface with 600 nm period and 110 nm radius. The size distribution of the particles is acquired from multiple SEM images using ImageJ software. The radius sizes are distributed within ±3nm (~ 3% deviation). The same deviation value is ±6nm (~ 4%) for LIL fabricated ones, proves the LIL like homogeneity of EBL fabricated structures.

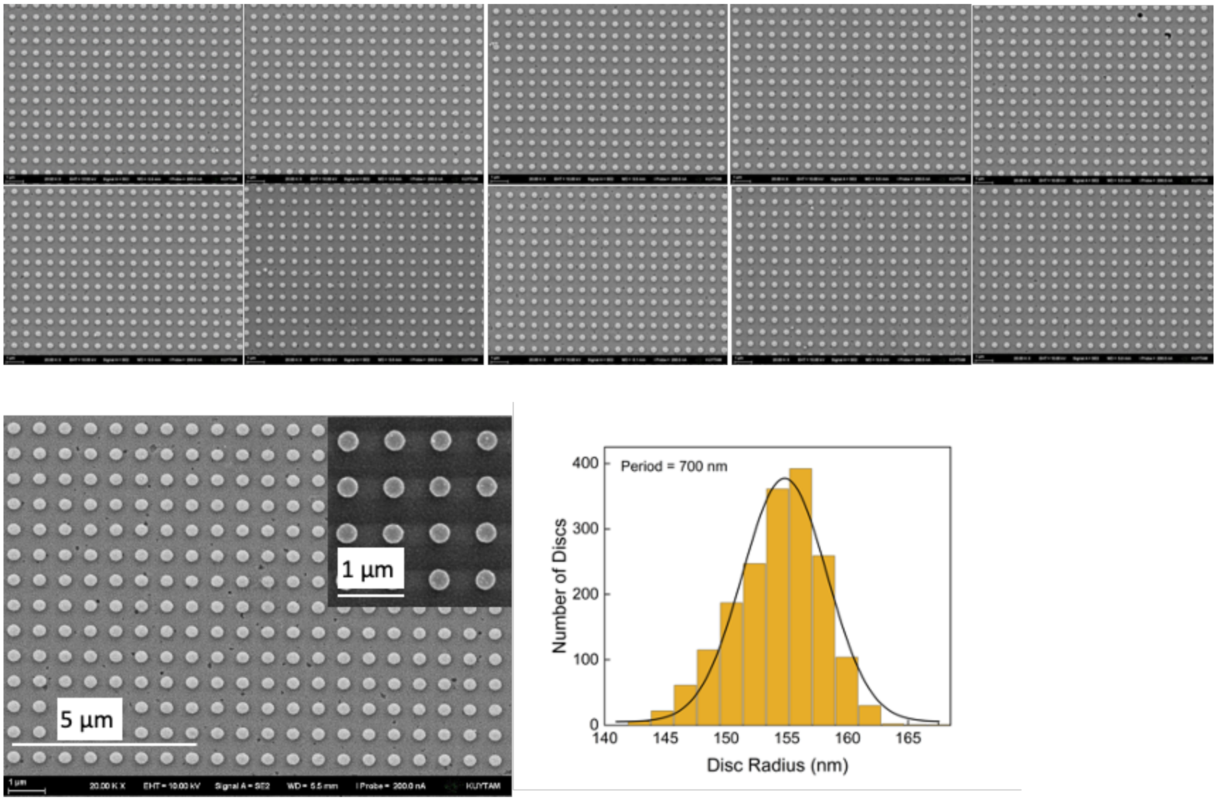

Figure S5. Ten SEM images on top are obtained from points of a LIL fabricated metasurface with P= 700 and R= 155nm (also used in Figure S1a). The size distribution of the particles is acquired using ImageJ software. The radius sizes are distributed within ~ ±6nm (~ 4% deviation).

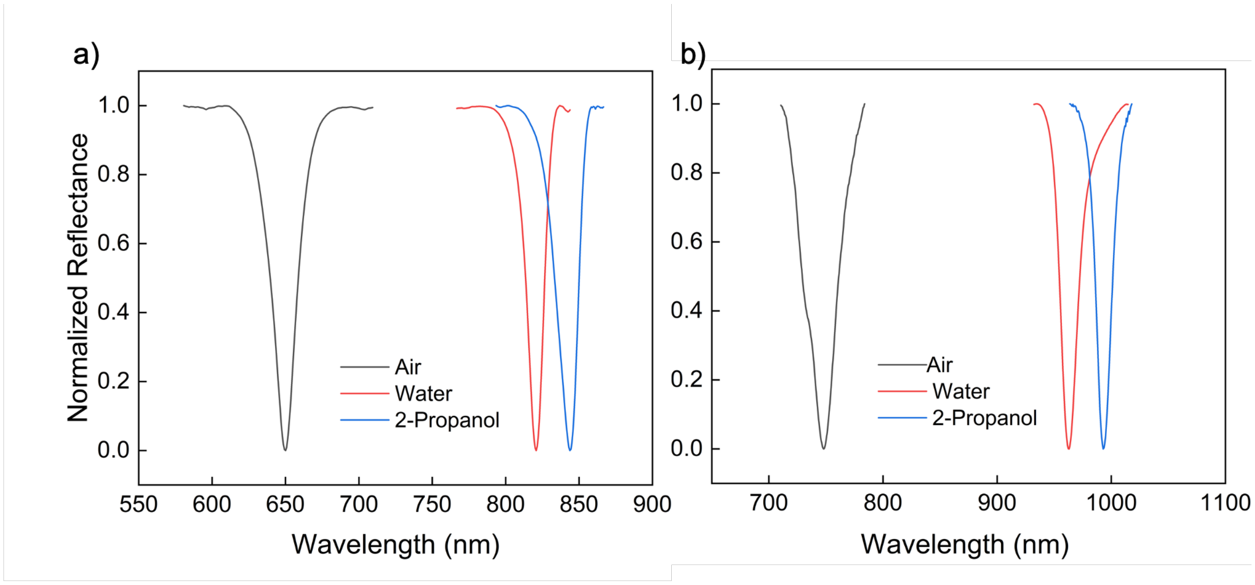

Figure S6. The reflection spectra of the EBL fabricated nanodisc based metamaterial surface with P=600 nm (a) and P=700 nm (b) are shown in air, water and 2-propanol. The sensitivity (S=Δλ/Δn) is calculated as 511nm/RIU for P=600 nm, and as 652 nm/RIU for P= 700 nm.

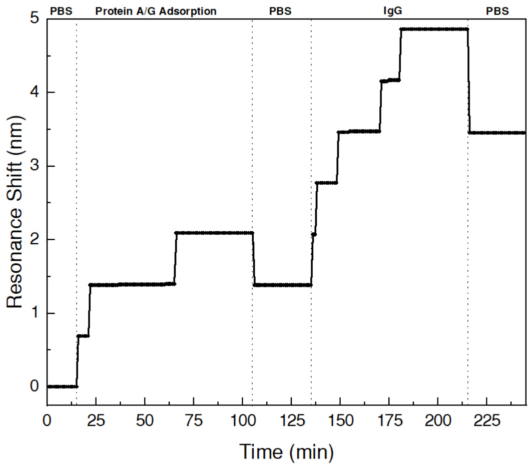

Figure S7. The raw data that is acquired during the real-time sensing of protein A/G and IgG. As shown in Figure 4a, we use a simple reflection probe with ~ 0.6 nm spectroscopic resolution. Even though the resolution is not high, the results clearly show the step-by-step resonance wavelength change during the protein A/G- IgG binding. We process this data by taking the average of 3 consecutive dots, and applied an exponential fitting to connect the dots, to obtain Figure 5b.

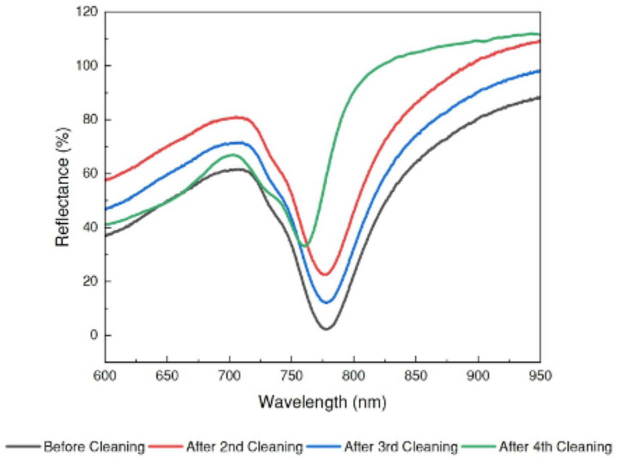

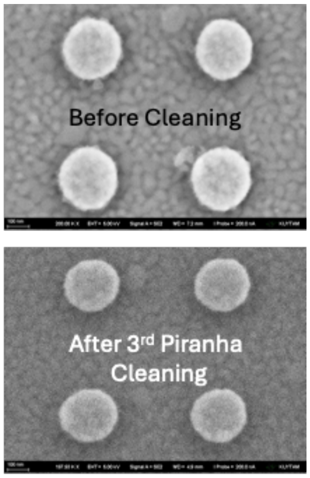

Figure S8. Piranha cleaning does not degrade the metasurface. The SEM images on the right shows the nanodiscs before any piranha cleaning and after 3rd piranha cleaning (H_2SO_4: H_2O_2, 3:1, for a minute). The corresponding optical responses are shown on the left. After the 3rd cleaning, the nanodiscs start to come off the surface and optical response deteriorates.

Table S1. Comparison of sensitivity S against previously reported values.

Reference	Simulation / Experiment	Wavelength Range	Device	Sensitivity (nm/RIU)
**1- This Work**	**Experimental**	**Vis-NIR**	**Metasurface based**	**685**
2	Simulation	Near IR	Metasurface based	460–492
3	Experimental	Near IR	Metasurface based	323
4	Simulation	Vis-NIR	Metasurface based	300
5	Simulation	Near IR	Metasurface based	551
6	Simulation	Near IR	Metasurface based	630.2
7	Experimental	Near IR	Metasurface based	400
8	Experimental	Mid IR	Metasurface based	372
9	Simulation	Near IR	Metasurface based	504
10	Simulation	Near IR	Metasurface based	840

[2] Khonina, S.N. et al. Numerical investigation of metasurface narrowband perfect absorber and a plasmonic sensor for a near-infrared wavelength range. *J. Opt.* **2021**, 23, 065102.

[3] Ollanik, A. J et al. Highly Sensitive, Affordable, and Adaptable Refractive Index Sensing with Silicon-Based Dielectric. *Adv. Mater. Technol.* **2019**, 4, 1800567.

[4] Vafapour, Z. et al. The Potential of Refractive Index Nanobiosensing Using a Multi-Band Optically Tuned Perfect Light Metamaterial Absorber. *IEEE SENSORS JOURNAL*. **2021**, 21, 12.

[5] Liang, J. et al. Multiband-switchability and high-absorptivity of a metamaterial perfect absorber based on a plasmonic resonant structure in the near-infrared region. *RSC Adv.*, **2022**, 12, 30871.

[6] Liang, C. et al. Dual-Band Infrared Perfect Absorber Based on a Ag-Dielectric-Ag Multilayer Films with Nanoring Grooves Arrays. *Plasmonics*. **2020**, 15:93–100.

[7] Liu, N. et al. Infrared Perfect Absorber and Its Application As Plasmonic Sensor. *Nano Lett.* **2010**, 10, 2342–2348.

[8] Le, K. Q. et al. Fabrication and Numerical Characterization of Infrared Metamaterial Absorbers for Refractometric Biosensors. *Journal of ELECTRONIC MATERIALS*, **2017**, 46, 1.

[9] Ali, W et al. Near-Infrared Perfect Absorption and Refractive Index Sensing Enabled by Split Ring Nanostructures. *Nanomaterials,* **2023**, 13, 2668.

[10] Lu, X. et al. Numerical investigation of narrowband infrared absorber and sensor based on dielectric-metal metasurface. *OPTICS EXPRESS,* **2018**, 26, 8, 10183.
